# Risk factors for familial clustering of hepatitis C virus infection in a Chinese Han population: a cross-sectional study

**DOI:** 10.1186/s12889-018-5592-5

**Published:** 2018-06-07

**Authors:** Bi-fen Luo, Hui-ying Rao, Ying-hui Gao, Lai Wei

**Affiliations:** Peking University People’s Hospital, Peking University Hepatology Institute, Beijing Key Laboratory of Hepatitis C and Immunotherapy for Liver Diseases, No. 11 Xizhimen South Street, Beijing, 100044 China

**Keywords:** Hepatitis C, Familial clustering, Risk factors

## Abstract

**Background:**

Hepatitis C is a curable disease, but reinfection from household contact may occur in patients who have achieved sustained viral response (SVR).

**Methods:**

A total of 997 ethnic Han HCV treatment-naïve adult patients were enrolled in a cross-sectional study with stratified sampling based on the populations of five geographic regions across China to examine the genetic and physiological parameters associated with the phenomenon of HCV familial clustering.

**Results:**

Of the total 997 patients, there were 59 patients who had at least one family member with HCV infection according to patient self-report. Comparison between patients with and without HCV familial clustering by univariate regression analysis showed that genotype 2, sexual transmission, long-term exposure to HCV patients, monthly family income per person less than 2000 yuan, farming occupation, and the southern and northern regions were associated with HCV familial clustering. Blood transfusion was negatively associated with HCV familial clustering. Multivariate logistic regression analysis suggested that long-term exposure to HCV patients and low family income were correlated with HCV familial clustering, whereas blood transfusion was negatively associated, which meant that blood transfusion was not the main transmission route in HCV familial clustering.

**Conclusion:**

Long-term exposure to HCV patients and low family income were correlated with HCV familial clustering, whereas blood transfusion was not the main transmission route in HCV familial clustering. To reduce reinfection from household contacts, education and awareness of HCV transmission routes and familial clustering should be strengthened, especially among HCV patients’ family members, low-income families and non-blood transmission hepatitis C patients.

## Background

Hepatitis C virus (HCV) is globally distributed, and it is estimated that up to 80 million persons have chronic HCV infection around the world [1]. Over the past two decades, the sustained viral response (SVR) rate of hepatitis C treatment has steadily increased. The World Health Organization(WHO) Global health sector strategy on viral hepatitis 2016–2021 is expected to deliver a 70% reduction in HCV incidence by 2030 compared with 2010 [2]. This is a rather challenging task. In the era of direct-acting antivirals (DAAs), the SVR rate has increased to more than 90% [3]. However, due to high prices, not all chronic hepatitis C patients have the opportunity to receive timely treatment with DAAs, especially in low-income areas. Additionally, even if hepatitis C has been cured, the immune response to HCV infection is inadequate to clear a subsequent exposure, and there is no effective vaccine for HCV currently [4]. Thus, persons who have achieved SVR are still at risk of HCV reinfection if they continue to be exposed to HCV transmission risk factors [5–11]. HCV familial clustering and intrafamilial viral transmission have been demonstrated previously despite the low rate of intrafamilial transmission [12–15]. Household contact with HCV patients is also a risk factor. Reinfection may occur in household settings if other family members are unaware of their infection and ongoing risk behaviors. To eradicate HCV, in addition to potent drugs, increasing awareness of HCV infection among family members of hepatitis C patients and changing lifestyle habits to avoid reinfection through household settings are essential.

Risk factors for HCV familial clustering are controversial. In some studies, HCV familial clustering was relevant for high HCV RNA levels, severe liver disease, advanced age, long-term contact, sharing of personal hygiene items, and having a spouse who was infected with HCV [12, 13, 16]. The relationship between siblings and offspring was also a risk factor in a few studies [17].

In China, there are some data concerning familial clustering from epidemiological surveys among restricted populations in areas with very high prevalence of anti-HCV, up to 28.9–43.4% [16, 18, 19]. The risk factors for familial clustering in Chinese ethnic Han HCV patients have not been properly explored. Moreover, there has been no research focused on the relationship between HCV familial clustering and socioeconomic factors.

This study investigated the risk factors for familial clustering of HCV among the Chinese Han population via a large cross-sectional observational study.

## Methods

### Patients

A total of 1012 ethnic Han HCV treatment-naïve patients ≥18 years old were enrolled in this study using stratified sampling based on populations from five geographic regions at 28 university-affiliated hospitals across China between February and June 2011. The Han ethnic group accounts for approximately 92% of the Chinese population. Provinces in China were grouped into five geographic regions (North, South, East, West, and Central). Patient enrollment and sampling were weighted according to the population density within each region. HCV infection was confirmed or reconfirmed by anti-HCV antibody and positive HCV RNA testing within 90 days prior to enrollment. Patients who had received antiviral or interferon-based therapy for hepatitis C or hepatitis B were excluded. There were no other exclusion criteria.

Of the 1012 participants, 15 patients were excluded for protocol violations (one patient had two violations). Two patients were < 18 years old and/or not ethnic Han. HCV infection was not confirmed within 90 days prior to enrollment for 10 patients. Four patients failed to undergo physical examinations and have their blood drawn within 9 days after providing informed consent. As a result, the cohort included a total of 997 patients.

### Definition of HCV familial clustering

If a patient had one or more family members known to be infected by HCV by self-report, information about their relationship with them and the timing of diagnosis of HCV infection were collected. Index cases were defined as patients with familial clustering of HCV. Other patients in the cohort were defined as controls.

### Clinical parameters

The clinical parameters of age, gender, self-reported HCV transmission risk factors, time of HCV diagnosis, possible duration of HCV infection, and socioeconomic factors, such as marriage, education, occupation and monthly family income, were measured and recorded. Laboratory examinations, such as those for liver enzymes, complete blood count, HCV polymerase chain reaction, and HCV genotype, were tested. Child-Turcotte-Pugh scores were calculated. An operator blind to the clinical and biological data of the patients was used for all tests and analyses.

### HCV-RNA quantitative analysis, HCV genotypes

The HCV viral load was tested using an Abbott Real-time HCV Genotype II (Abbott Laboratories, Des Plaines, IL, USA). Six different genotypes of viral HCV were assessed using a Versant HCV Genotype 2.0 Assay (LiPA) by Siemens, according to the manufacturer’s instructions (Siemens Healthcare Diagnostics, Tarrytown, NY, USA).

### Statistical analysis

Categorical variables were tabulated with counts and percentages; continuous variables were summarized with medians and quartiles due to their non-normal distributions. Odds ratios (OR) and 95% confidence intervals (CI) for the factors under consideration were calculated in univariate and multivariate logistic regression analysis. *P* < 0.05 was considered to be statistically significant.

## Results

### Patient characteristics

Of the total 997 patients, there were 59 patients with familial clustering of HCV. The other 938 patients were assigned to the control group. The basic characteristics of the 59 patients are listed in Table 1. Thirty patients (50.8%) were male. The median age was 43 years (Q1 = 37, Q3 = 53). There was only one patient with cirrhosis. The median diagnosis time was 5 months (Q1 = 1, Q3 = 24), with a median duration of possible infection of 203 months (Q1 = 165, Q3 = 244). The median HCV RNA was 6.06 Log_10_IU/ml (Q1 = 5.17, Q3 = 6.53). The characteristics between the two groups were not significantly different with regard to gender, age, cirrhosis, diagnosis time, duration of possible infection and HCV RNA level. Among patients with familial clustering of HCV, 39.0, 39.0, 10.2, and 8.5% of patients were infected by hepatitis C viral genotypes 1, 2, 3, and 6, respectively. Among controls, patients with hepatitis C viral genotypes 1, 2, 3, and 6 comprised 59.6, 23.1, 9.1 and 6.2%, respectively. The prevalence of the HCV genotype 2 was higher in patients with familial clustering of HCV according to univariate logistic regression analysis (OR = 2.576, *P* = 0.002).

**Table 1:** Patient demographics and liver disease factors predicting the presence or absence of familial clustering of HCV using univariate logistic regression analysis

Parameters	Familial Clustering		OR	95%CI		*P* value
	Yes *n* = 59 (%)	No *n* = 938(%)		L	U	
Gender			0.846	0.500	1.432	0.534
Male	30(50.8)	516(55.0)				
Female	29(49.2)	422(45.0)				
Age, median years (Q1,Q3)	43(37,53)	46(37,56)	0.990	0.971	1.010	0.338
Cirrhosis			0.144	0.020	1.054	0.056
Yes	1(1.7)	100(10.7)				
No	58(98.3)	838(89.3)				
Diagnosis time, median months (Q1,Q3)	5(1,24)	3(1,28)	1.002	0.998	1.007	0.280
Possible infected duration, median months (Q1,Q3)	203(165,244)	219(160,252)	0.999	0.996	1.002	0.593
HCV RNA, median Log_10_IU/ml (Q1,Q3)	6.06(5.17,6.55)	6.07(5.37,6.53)	1.030	0.788	1.347	0.826
Genotype						
1	23(39.0)	559(59.6)	1.000			
2	23(39.0)	217(23.1)	2.576	1.415	4.688	0.002*
3	6(10.2)	85(9.1)	1.716	0.679	4.335	0.254
6	5(8.5)	58(6.2)	2.095	0.768	5.719	0.149
Mixed	2(3.4)	19(2.0)	2.558	0.562	11.645	0.224

### Route of HCV transmission

The possible infection routes are listed in Table 2. The most common infection route was long-term exposure to HCV patients among patients with familial clustering of HCV (37.3%), and it was blood transfusion in the control group (59.5%). Sexual transmission and long-term exposure to HCV patients were found to have the highest correlations with familial clustering of HCV, and blood transfusion was negatively correlated with familial clustering of HCV according to univariate logistic regression analysis (OR = 5.336, 185.318, 0.279 and *P* = 0.002, < 0.001, < 0.001, respectively). The incidence of infection routes via intravenous drug abuse, intravenous infusion, dental treatment, tattooing and piercing, blood purification, intra-examination/ treatment, surgery and transplantation was not significantly different between patients with familial clustering of HCV and controls. No mother-to-child transmission occurred in either group.

**Table 2:** Infection routes predicting the presence or absence of familial clustering of HCV using univariate logistic regression analysis

Parameters	Familial Clustering		OR	95%CI		*P* value
	Yes *n* = 59 (%)	No *n* = 938(%)		L	U	
Sexual transmission			5.336	1.884	15.111	0.002*
Yes	5(8.5)	16(1.7)				
No	54(91.5)	922(98.3)				
Blood transfusion			0.276	0.155	0.491	< 0.001*
Yes	17(28.8)	558(59.5)				
No	42(71.2)	380(40.5)				
Intravenous drug abuse			1.131	0.438	2.917	0.799
Yes	5(8.5)	71(7.6)				
No	54(91.5)	867(92.4)				
Intravenous Infusion			0.732	0.223	2.402	0.606
Yes	3(5.1)	64(6.8)				
No	56(94.9)	874(93.2)				
Dental treatment			1.064	0.527	2.146	0.863
Yes	10(16.9)	151(16.1)				
No	49(2.9)	787(83.9)				
Long-term exposure to HCV patients			185.315	53.082	646.951	< 0.001*
Yes	22(37.3)	3(0.3)				
No	37(62.7)	935(99.7)				
Tattooing and piercing			1.600	0.662	3.866	0.297
Yes	6(10.2)	62(6.6)				
No	53(89.8)	876(93.4)				
Blood purification			0.000	0.000	0.000	0.999
Yes	0(0)	15(1.6)				
No	59(100)	923(98.4)				
Intra-exam/treatment			1.342	0.401	4.494	0.633
Yes	3(5.1)	36(3.8)				
No	56(94.9)	902(96.2)				
Surgery and transplantation			0.713	0.345	1.476	0.363
Yes	9(15.3)	189(20.1)				
No	50(84.7)	749(79.9)				

### Socioeconomic factors

Monthly family income of less than 2000 Yuan per person and farming occupation were significantly correlated with familial clustering of HCV (OR = 2.207, 2.087, *P* = 0.008, 0.013, respectively). Compared to the central region, there were more patients with familial clustering of HCV in the southern and northern regions (OR = 3.591, 2.731, *P* = 0.007, 0.034, respectively). The parameters of marriage and education were not significantly correlated with HCV familial clustering (Table 3).

**Table 3:** Socioeconomic factors predicting the presence or absence of familial clustering of HCV using univariate logistic regression analysis

Parameters	Familial Clustering		OR	95%CI		*P* value
	Yes *n* = 59 (%)	No *n* = 938(%)		L	U	
Marriage			0.690	0.270	1.761	0.435
Married	54(91.5)	827(88.2)				
Single	5(8.5)	111(11.8)				
Monthly family income/ person			2.207	1.226	3.975	0.008*
< 2000	43(72.9)	515(54.9)				
≥ 2000	16(27.1)	423(45.1)				
Education						
Illiteracy/Semiliterate	4(6.8)	46(4.9)	1.000			
Primary school	12(20.3)	115(12.3)	1.200	0.368	3.914	0.762
Junior school	17(28.8)	254(27.1)	0.770	0.248	2.391	0.651
High school	13(22.0)	260(27.7)	0.575	0.180	1.841	0.351
Associate	7(11.9)	130(13.9)	0.619	0.173	2.213	0.461
College	6(10.2)	116(12.4)	0.595	0.160	2.205	0.437
Graduate	0(0)	17(1.8)	0.000	0.000	–	0.998
Occupation			2.087	1.167	3.726	0.013*
Farming	18(30.5)	163(17.4)				
Other	41(69.5)	775(82.6)				
Region						
East	14(23.7)	217(23.1)	2.101	0.832	5.305	0.116
West	10(16.9)	199(21.2)	1.637	0.612	4.380	0.327
South	14(23.7)	127(13.5)	3.591	1.413	9.127	0.007*
North	14(23.7)	167(17.8)	2.731	1.078	6.914	0.034*
Central	7(11.9)	228(24.3)	1.000			

### Multivariate logistic regression analysis

**Fig. 1:**
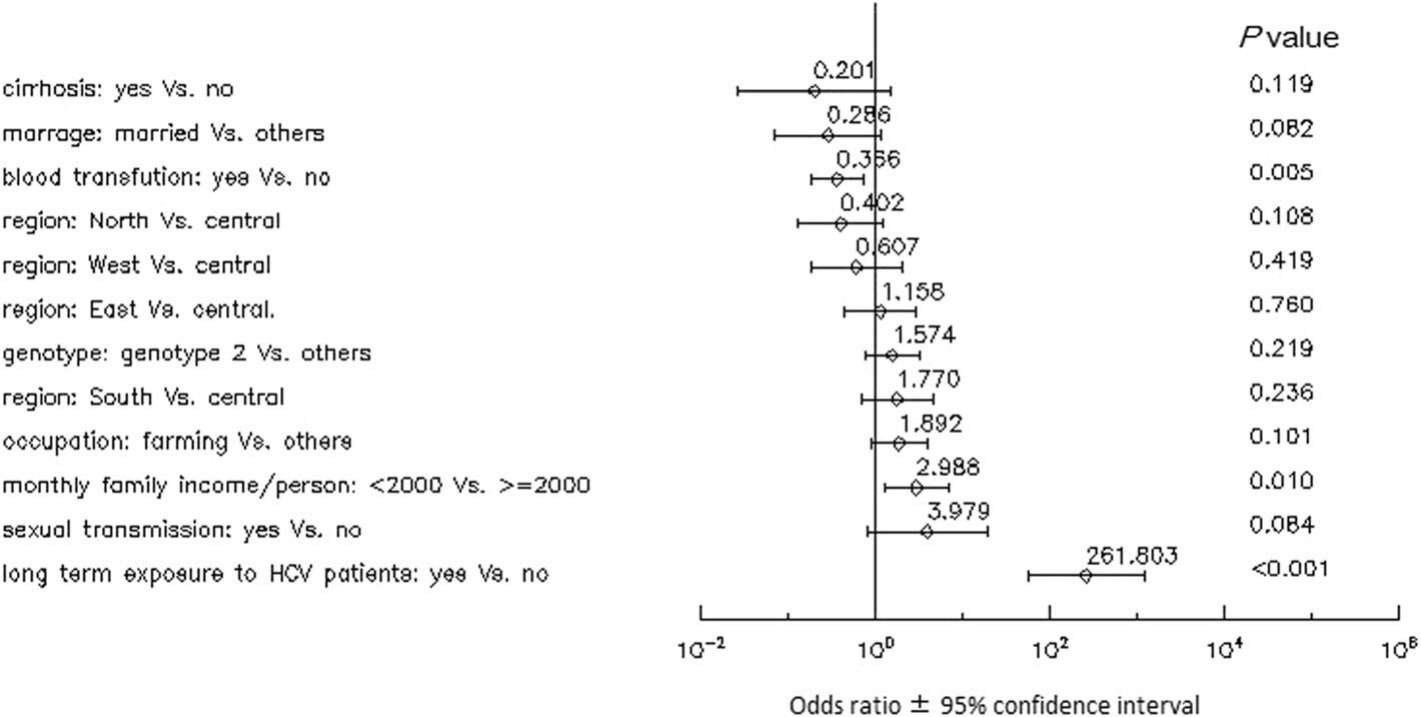
Correlates of HCV familial clustering. Odds ratios and 95% confidence intervals are shown for parameters under consideration in the multivariate model

Multiple logistic regression analysis suggested that long-term exposure to HCV patients and low family income were associated with HCV familial clustering, whereas blood transfusion was negatively associated with it (Fig. 1). This suggests that blood transfusion was not the main transmission route in HCV familial clustering.

## Discussion

Parenteral routes, such as blood transfusion, intravenous drug use and invasive medical procedures, are the primary infection routes for HCV transmission. However, there has been much debate regarding other potential modes of transmission, particularly as a substantial proportion of HCV cases do not have a defined parenteral exposure [15]. Epidemiological evidence for intrafamilial transmission relies on the higher prevalence of anti-HCV/ HCV RNA positivity among household contacts compared with the general population [12–14, 16]. One study in Egypt showed that the percentage of HCV infections was higher among family members of HCV-positive index cases than among family members of HCV negative-index cases, 38/257, 14.8% Vs. 3/75, 4% [20]. Phylogenetic analysis of HCV sequences showed homology between index cases and household contacts, confirming intrafamilial transmission of HCV [15], despite the low effectiveness [19].

Previous studies have suggested the following risk factors for HCV familial clustering: long-term relationship with index cases, long-term marriage with index cases [21], advanced age [20], index cases with severe liver disease (cirrhosis and liver carcinoma) [22], family members who consume alcohol [23], having a history of intravenous drug abuse or incarceration, blade/needle sharing [24], having contact with patients with hepatic encephalopathy [20], and even first-degree relatives, based on genetic predisposition [17]. The current study confirmed that long-term exposure to HCV patients was associated with familial clustering of HCV. This study also showed that low family income was a risk factor, and blood transmission was negatively associated with familial clustering of HCV.

In household settings, family members have more opportunities to be exposed to the blood and body fluid, such as saliva and sperm, of index cases, which has been demonstrated to be HCV RNA-positive [25], resulting in the risk of intrafamilial transmission.

In low-income areas, sharing hygienic products, such as shaving razors, toothbrushes, and nail clippers is common due to ignorance about sanitation and lack of resources. Sharing hygienic items is an important risk factor for HCV transmission [16, 24]. As mentioned, in low-income areas, DAAs treatment may not be available for reasons of expense, which makes it more important to raise awareness about the facts of HCV intrafamilial transmission.

Compared with the control group, blood transmission was a less common route for infection in the familial clustering group, perhaps because the majority of familial clustering patients were infected with HCV through household contact rather than blood transfusion.

Sexual transmission has been proven to be one type of transmission route for HCV [13, 16]. In most studies, spousal relationship with an index case has been found to be a risk factor for intrafamilial transmission of HCV. In this study, sexual transmission was more common in patients with familial clustering of HCV according to univariate logistic regression analysis, but the difference was not significant according to multivariate logistic regression analysis. Unlike HBV infection, mother-to-child transmission was relatively uncommon in HCV patients, with a risk of approximately 4–8% [26, 27]. In this study, mother-to-child transmission was not suspected in any of the 997 patients with HCV.

In this study, hepatitis C-related factors, such as HCV RNA, genotype, cirrhosis, time of diagnosis, and time of first potential exposure to HCV, were not associated with HCV familial clustering. Although genotype 2a was more common in the group with familial clustering than in the control group according to univariate analysis, the difference was not significant in the multivariate model. Piao et al. reported that familial clustering of HCV existed in the Yanbian Prefecture in northern China. Using a case-control study, 200 anti-HCV-positive and HCV RNA-positive patients and 200-HCV negative controls were examined. In total, 49 family members of HCV index cases and 19 family members of controls were enrolled. They found that intrafamilial transmission of HCV infection was mainly through sexual activity between spouses, and there was no statistically significant relationship with HCV RNA or genotype. Similar to our study, genotype 2a was more common in family members in the HCV group than in the non-HCV group, but this difference was not statistically significant [16].

Other than low family income, socioeconomic factors such as marriage, education, and occupation were not associated with HCV interfamilial clustering.

Reinfection with HCV can occur after successful antiviral treatment [6, 28], with spontaneous clearance among high-risk groups [9, 29]. The reinfection rate varied from very low (0–5 cases per 100 person-years) [28, 30] to as high as 24.6 cases per 100 person-years [31] among injecting drug users (IDUs) and 9.4 cases per 100 person-years in HIV-positive men who have sex with men (MSM) [8]. There is little data regarding reinfection in a population with familial clustering of HCV let alone in large prospective analyses. There are some difficulties with such an investigation. To design a clinical trial on reinfection from household contracts, it should contain HCV detection and regular follow-up examinations, not only for SVR index cases but also for their family members. Once family members are aware of their disease, they will probably seek treatment to cure them of hepatitis C, resulting in a reduced rate of HCV infection status in family members than would otherwise be seen naturally. Due to low rates of intrafamilial transmission of HCV, such a study might require a large-scale sample and long-term follow-ups. When reinfection occurs, genotyping or sequencing would be needed to distinguish reinfection from late relapse. And collecting information on behavior to exclude other reinfection routes would be required.

Based on evidence of intrafamilial transmission of HCV, presumably, reinfection may occur in household settings. However, the public knows little about the intrafamilial clustering of HCV and risk factors. As such, individuals who have achieved SVR may not change their daily habits, for instance, sharing hygienic items. As a consequence, they may still be at risk of reinfection. It is urgent to provide public health education regarding HCV, including family clustering, risk factors, and transmission routes of HCV, especially for individuals who have achieved SVR.

Some of the advantages of this study include its large-scale sample of approximately 1000 patients, the large area coverage, which included five geographic regions across the entire country, and the consideration of socioeconomic factors. There are also a few limitations, for instance, self-report of HCV infection in family members without laboratory confirmation following enrollment, as well as lack of information about the total number of family members and the percentage of HCV-infected members.

## Conclusion

The current study suggests that long-term exposure to HCV patients and low family income are risk factors for familial clustering of HCV. Long-term exposure to HCV patients and low family income were correlated with HCV familial clustering, whereas blood transfusion was not the main transmission route in HCV familial clustering. To reduce the reinfection rate from household contacts, education on and awareness of HCV transmission routes and familial clustering should be strengthened, especially among family members of HCV-infected patients, low-income families and non-blood transmission hepatitis C patients.

## Abbreviations

CI: Confidence interval
DAAs: Direct-acting antivirals
HCV: Hepatitis C virus
OR: Odds ratio
SVR: Sustained viral response
WHO: The World Health Organization
